# Central nervous system tumours in neonates: what should the neonatologist know?

**DOI:** 10.1007/s00431-023-05404-3

**Published:** 2024-01-11

**Authors:** Maristella Toniutti, Annalisa Lo Sasso, Andrea Carai, Giovanna Stefania Colafati, Eleonora Piccirilli, Giada Del Baldo, Angela Mastronuzzi

**Affiliations:** 1 Department of Medicine DAME-Division of Pediatrics, University of Udine, Udine, Italy; 2 Department of Neurosciences, Neurosurgery Unit, Bambino Gesù Children’s Hospital, IRCCS, Rome, Italy; 3 Department of Diagnostic Imaging Oncological Neuroradiology Unit, Bambino Gesù Children’s Hospital, IRCCS, Rome, Italy; 4 Department of Neuroscience, Imaging and Clinical Sciences, University “G. d’Annunzio” of Chieti-Pescara, Chieti, Italy; 5 Department of Pediatric Haematology and Oncology, and Cell and Gene Therapy, Bambino Gesù Children’s Hospital, IRCCS, Rome, Italy; 6 Department of Experimental Medicine, Sapienza University of Rome, Rome, Italy

**Keywords:** Brain tumours, Newborn, Congenital CNS tumour, Fetal tumour

## Abstract

Central nervous system (CNS) tumours in neonates are relatively rare and present differently when compared with those occurring later in childhood in terms of aetiology, clinical features, location, histology and prognosis. The clinical presentation is extremely variable. Even if the most frequent clinical sign is a macrocephaly, there are many other non-specific symptoms associated. The prognosis is usually poor with overall survival of less than 30%. Surgery continues to be the primary treatment for neonatal CNS tumours, aiming for a gross total resection, directly correlated with prognosis and the overall outcome. The chemotherapy is the only adjuvant therapy whereas the radiotherapy is avoided under three years of age because of the severe sequelae*.* Hence the importance of molecular characterization of these neoplasms in order to improve the accuracy of the diagnosis and identify new therapeutic targets. The aim of this review is to describe the main characteristics of these tumours and the recent advances in their treatment in order to recognize these pathologies in the prenatal period and create a multidisciplinary team providing the best possible treatment while minimising the risk of long-term complications. Neonatologists play a key role in the early detection, diagnostic evaluation, management and supportive care of these neonates.

*Conclusion*: The aim of this review is to describe the main characteristics of these tumours and the recent advances in their treatment in order to ensure the essential knowledge that will help the neonatologist identify them and create a multidisciplinary team providing the best possible treatment while minimising the risk of long-term complications.

**Table:** 

**What is Known:** *• Neonatal CNS tumours are relatively rare and their early identification is important to identify the best diagnostic-therapeutic management.* *• Surgery is the main treatment of neonatal CNS tumours. The extent of surgical resection directly correlates with prognosis and outcome.*	
**What is New:** *• Predisposing conditions such as Cancer Predisposition Syndromes must be considered.* *• Targeted drugs and other therapeutic strategies can be identified through molecular characterization*

**What is Known:**

*• Neonatal CNS tumours are relatively rare and their early identification is important to identify the best diagnostic-therapeutic management.*

*• Surgery is the main treatment of neonatal CNS tumours. The extent of surgical resection directly correlates with prognosis and outcome.*

**What is New:**

*• Predisposing conditions such as Cancer Predisposition Syndromes must be considered.*

*• Targeted drugs and other therapeutic strategies can be identified through molecular characterization*

## Introduction

Central nervous system (CNS) tumours are the primary cause of cancer-related mortality in children, making them significant pathologies. This is particularly true for infants, where the rarity of the disease contributes to limited data availability.

An early suspicion and the initiation of an appropriate course of treatment are in most cases crucial to change the outcome of these patients. This has become more and more important over the years, owing to advances in histopathological, and above all, molecular characterization, which can sometimes give therapeutic chances for tumours previously considered untreatable.

The aim of our work is to review the main characteristics of these tumours in order to provide the essential knowledge that will help the neonatologist identify them and subsequently administer personalised treatments.

## Epidemiology

CNS tumours are the second most frequent neoplasms in childhood after leukaemia, first among solid tumours. They account for 0.5–1.9% of all malignant tumours in children [1, 2], but the prevalence increases if we consider only the infant stage (< 5 years) [3].

The incidence of childhood CNS tumours varies from 1.12 to 5.14 cases per 100,000 people [4]. These differences may stem partly from unequal diagnostic capabilities, often resulting in lower incidence in developing countries, and partly from a lack of paediatric data and national registries. The underreported foetal prevalence due to stillbirths or in utero deaths and differences in infant definition must be also taken into account [3]*.*

## Causes/risk factors

Neonatal CNS tumours are relatively rare, and their exact causes are not well understood although a multifactorial aetiology is accepted. There are several factors that may contribute to their development.

### Genetic factors

The main etiological factor related to the presence of congenital neoplasms is the presence of a Cancer Predisposition Syndrome (CPS) [4], the identification of a variant, de novo or inherited, that causes a genetic syndrome.

Table 1 summarizes the main syndromes related to the onset of brain tumours.

**Table 1:** Cancer Predisposition Syndromes [5–9]

***SYNDROMES***	***GENE***	***ASSOCIATED TUMOUR TYPE***
Li-Fraumeni syndrome	TP53	All gliomas, choroid plexus carcinoma
Neurofibromatosis Type 1 Type 2	*NF1* *NF2*	Astrocytoma, neurofibroma (Fig. 1), schwannomas, optic nerve glioma, acoustic neuromas, meningiomas, spinal ependymomas
Tuberous Sclerosis Complex (TSC)	*TSC1, TSC2*	Subependymal giant cell astrocytoma
Turcot syndrome Type 1 Type 2	MLH1, PMS2 APC	Medulloblastoma, glioblastoma
Gorlin syndrome	PTCH1	Medulloblastoma, meningioma
Cowden syndrome	PTEN	Cerebellar gangliocytoma, meningioma
Lynch syndrome	MSH2, MSH6, MLH1, PMS2	Medulloblastoma, gliomas
Rhabdoid tumour predisposition syndrome (RTPS)	SMARCB1, SMARCA4	ATRT, medulloblastoma, choroid plexus tumours
Von Hippel-Lindau syndrome	VHL	Hemangioblastoma
Retinoblastoma	RB1	Retinoblastoma, pineoblastoma, malignant glioma
Ataxia-telangiectasia	ATM	Astrocytoma and medulloblastoma
DICER1 syndrome	DICER1, DROSHA	Pineoblastoma, pituitary blastoma
Rubinstein–Taybi syndrome	CREBBP, EP300	Medulloblastoma, oligodendroglioma, and meningioma
Fanconi Anaemia	FANCA	Medulloblastoma

Detailed genetic testing should be recommended in all cases of neonatal cancer. This allows for better diagnostic and therapeutic planning, and screening of the proband’s siblings. In these cases, a meticulous search of additional foetal anomalies is crucial for prenatal–postnatal management and parental counselling.

From the point of view of family history, the presence of a first-degree relative with a brain tumour is ambiguous [4]. However, the presence of several neoplasms in the parental lineage should raise the suspicion of CPS and is worthy of further investigation.

Advanced parental age, particularly paternal, may be a risk factor for congenital cancers as a marker of inherited somatic changes [4, 11, 12]*.*

### Environmental factors

Prenatal exposure to certain environmental factors may play a role in the development of central nervous system tumours, although the strength of evidence is not very high.

There appears to be a positive association between the risk of brain tumours and exposure to pesticides [13, 14]. However, the data are not always consistent and replicable.

Some infections during pregnancy, such as cytomegalovirus (CMV) or rubella, have been linked to an increased risk of CNS abnormalities, and in rare cases tumours [15].

## Signs and symptoms

The clinical presentation of neonatal CNS tumours is extremely variable. According to the onset of symptoms, most authors define neoplasms as definitely congenital if symptoms appear in the prenatal period or at birth, probably congenital if symptoms appear within the first week of life, and possibly congenital if symptoms appear within six months of birth [16, 17].

### Foetal presentation

Routine foetal ultrasound during pregnancy allows early detection of abnormalities or red flags that may denote the presence of a congenital intracranial mass and provides an indication for more advanced diagnostic tests.

The first sign of a congenital tumour with prenatal onset is usually polyhydramnios, more commonly in the second and third trimester secondary to both hypothalamic dysfunction and high cardiac output [2, 18].

The second most common condition is macrocephaly [19], resulting from either the growth of an intracranial mass or secondary to hydrocephalus caused by ventricular obstruction or increased production of cerebrospinal fluid, or even intracranial haemorrhage from the tumour itself [20]. In all cases, normal brain development may be impaired.

When a sizeable congenital brain tumour leads to hydrops or miscarriage, the diagnosis is made post-mortem.

### Neonatal presentation

The diagnosis may also be suspected in the neonatal period because of complications during labour, particularly cephalopelvic disproportion, which can lead to dystocia because of large foetal skulls [21]. A caesarean section is required in 60% of cases [22].

Even in neonatal onset, the main sign of a neonatal brain tumour is macrocephaly (50–60%) [23], which is often asymptomatic due to the plasticity of the neonatal skull. Nevertheless, elevated intracranial pressure can manifest as bulging fontanelles, irritability, lethargy, even apnoeic episodes and seizures [24]. Rarely, large and highly vascular masses may present with high-output heart failure.

### Infant presentation

In contrast, postnatal onset tends to be more insidious, with ongoing presence of macrocephaly or exponential growth of the head circumference, which may be associated with bulging fontanelles or delayed fusion of sutures.

Presentation can be with other non-specific symptoms such as drowsiness, irritability, vomiting, apnoea, developmental delay, lack of growth, or abnormal eye movements.

More acute onset is generally related to complications such as tumour bleeding or seizures [17].

## Diagnosis

As for the clinic, diagnosis can be differentiated between a prenatal phase and neonatal/postnatal one. In both cases, the presence of a mass is suspected due to suggestive clinic and/or ultrasound abnormalities, but further assessment is needed such as magnetic resonance imaging (MRI).

However, confirmation through histology is essential.

### Prenatal diagnosis

The first report of a brain tumour found in a 28-week gestation foetus by maternal ultrasound was documented by Hoff in 1980 [25]. Since then, resolutions and imaging techniques have improved exponentially, resulting in earlier and more precise detection of intracerebral neoplasms.

Prenatal ultrasound may reveal the presence of an intracranial mass, which can appear as a solid, cystic or calcified lesion [26, 27]. Alternatively, indirect signs such as macrocephaly and hydrocephalus can be observed. In such conditions, a foetal MRI is generally recommended. This diagnostic procedure enables a more detailed characterization of the lesion, including its location and adjacent anatomical structures [28].

Detecting these pathologies in the prenatal period has several advantages: first of all, it enables to carry out multidisciplinary parental counselling involving not only neonatologists and obstetricians but also those implicated in postnatal management such as oncologists and neurosurgeons; it additionally permits the planning of elective delivery, so as to prepare a medical-nursing team ready to reduce the risk of maternal and foetal complications; finally, given the poor prognosis, it also grants the possibility of interrupting pregnancy within legal terms.

### Postnatal diagnosis

In the neonatal period, transfontanellar ultrasound is usually the primary diagnostic step. This may be conducted routinely as for preterm infants or as a response to diagnostic suspicion. Also in this setting, ultrasound can directly observe intracranial masses or indicate their presence through indirect signs, such as hydrocephalus. The tumour typically appears as a neoformation with a heterogeneous pattern that the larger it is, the more it subverts the normal anatomy of the brain.

The diagnostic gold standard remains MRI, which in addition to confirming the presence of the lesion and defining its size and characteristics, can identify the presence of calcifications, adipose tissue, blood or other characteristics that may lead to a specific histotype [26]. Calcifications would be better visible in CT (Computed Tomography) scans [29]; however, this imaging technique is not used in neonatal setting due to the risk of radiation exposure.

The neonatal MRI procedure requires the infant to remain motionless for a long period of time, which is generally achieved through sedation by the neonatologist. Given the high anaesthesiological risk that some of these patients may have (e.g. bulbar tumour), non-pharmacological measures like “feed- and-sleep” method may be considered [30]. In addition to accurate characterisation, MRI is indispensable for planning neurosurgical intervention.

Actually, imaging only raises suspicion, but definitive diagnosis requires histopathological confirmation by biopsy.

Alongside histopathological analysis, molecular characterisation of the tumour is becoming increasingly crucial because of the potential for targeted therapy.

As a complement and depending on the suspected histotype, tumour markers can be measured, and diagnostic and staging lumbar puncture may be performed.

## Histological subtypes and molecular characterization

There may be a difference in the incidence of certain tumour types in neonates compared to older infants. Some commentators have speculated that this difference represents different aetiologies of tumourigenesis in neonates compared to older children. In addition to the different aetiologies, there are also differences in terms of clinical presentation, location and prognosis.

For example, regarding anatomical distribution, the majority of congenital CNS tumours are supratentorial, whereas paediatric tumours are more commonly infratentorial [19].

The gestational age can orient toward a specific histological diagnosis; for example, teratomas and hamartomas generally develop before the 22nd week, germ cell tumours between the 22 ^nd^ and the 32 ^nd^ weeks and astrocytomas and glioblastomas after the 32 ^nd^ weeks [31].

The table below (Table 2) summarises the main characteristics of histological subtypes in terms of location, histological findings and radiological appearance.

**Table 2:** Main characteristics of histological subtypes [5, 20, 32, 33]

***HISTOTYPES***	***LOCATION***	***HISTOLOGICAL FINDINGS***	***RADIOLOGICAL APPEARANCE***
*Teratoma*	Intracranial: -pineal or suprasellar region (common) -infratentorial (less often) Extracranial: -sacrococcygeal region -head and neck region	-Mature teratomas: large, heterogeneous cystic/solid masses with calcification and fatty tissue -Immature teratomas: less defined margins, fewer cysts and calcifications	Mixed-density lesion on CT, hypodense fatty components, coarse calcification (bone and teeth)
*Glioma:*			
*-LGG*	Supratentorial (optic nerve and hypothalamic region)	Neuroglial tumours composed by astrocytes, oligodendrocytes, and mixed oligoastrocytoma cells	Hypointense on T1-weighted images and hyperintense on T2 weighted images with marked and homogeneous contrast enhancement
*-HGG*	Supratentorial	Densely cellular tumours with marked pleomorphism, mitotic activity, vascular thrombosis, microvascular proliferation and necrosis	Isointense to the brain or slightly hyperintense on T1-weighted images and isointense on T2-weighted images. Restricted diffusion is typical
*Choroid plexus tumours*	Supratentorial (preferentially in the lateral ventricles)	Highly vascular tumours composed of mature epithelial cells	Intraventricular mass with homogeneous contrast enhancement; isointense to hypointense on T1-weighted images, isointense to hyperintense on T2-weighted images Hydrocephalus (common)
*Embryonal tumours:*			
*-Medulloblastomas (MB)*	Infratentorial (posterior cranial fossa)	The most common is MB with extensive nodularity, characterised by a macroscopic grape-like multinodular structure	Heterogeneous intensity on T1 and T2-weighted images; sparse calcification; restricted diffusion
*-ATRTs*	Infratentorial (common) Supratentorial (less often)	Immature rhabdoid cells, large tumour cells with vesicular nuclei, prominent red nucleoli, and moderate amounts of eosinophilic cytoplasm	Heterogeneous mass (solid components with restricted diffusion, isointense to hypointense on T2-weighted images)
*-Other embryonal tumours*	Supratentorial (common in cerebral hemispheres)	Undifferentiated or poorly differentiated neuroepithelial cells	Large heterogeneous mass hypointense on T2-weighted images; restricted diffusion
*Craniopharyngiomas*	Suprasellar region	Heterogeneous histology, large cystic masses which often calcify	Solid components (iso-hyperintense on T1-weighted images, variable signal intensity on T2-weighted images); cystic components (hypointense to hyperintense on T1-weighted images, hyperintense on T2-weighted images)
*Ependymomas*	Infratentorial (posterior cranial fossa) Supratentorial (cerebral hemispheres)	Ependymal cells composed of rather uniform darkly staining cells forming ependymal perivascular rosettes	Large multilobulated mass with intrinsic calcifications

Teratomas (Fig. 2) are the most common neonatal brain tumour, accounting for between a third and a half of all congenital tumours.

**Fig. 1:**
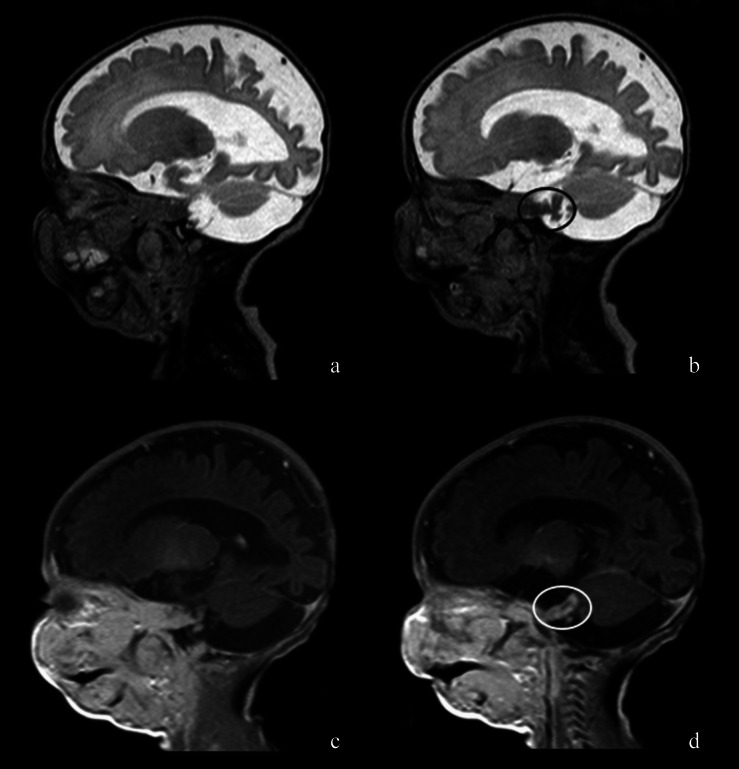
*Giant plexiform neurofibroma* of the right hemiface with intracranial component in a newborn with confirmed NF1 [10]. Sagittal T2 TSE (**a** and **b**) and post-contrast T1 images (**c** and **d**) show the intracranial extension along the trigeminal divisions to ipsilateral Meckel’s cave and intracisternal segments (circles)

Following teratomas, the most common neonatal CNS tumours are gliomas (18–47%) and choroid plexus tumours (5–20%). Among gliomas, we differentiate high-grade gliomas (HGG) and low-grade gliomas (LGG).

In addition to the histological features, molecular characterization is also important to improve the accuracy of the diagnosis. In fact, some neonatal brain tumours are associated with specific genetic mutations or alterations.

Tumours reported as infant HGG show significantly better survival than those in older children, suggesting that histopathological classification may not be representative of clinical behavior [34]. As such, they were classified separately in the current WHO 2021 classification [35]. This may be related to the fact that they have a distinct molecular profile, with fusion genes involving ALK, ROS1, NTRK1/2/3 or MET. For example, gene fusions involving the neurotrophin receptor tyrosine kinase genes 1–3 (NTRK1, NTRK2 and NTRK3) have been identified in 40% of non-brainstem HGGs in infants (Fig. 3).

**Fig. 2:**
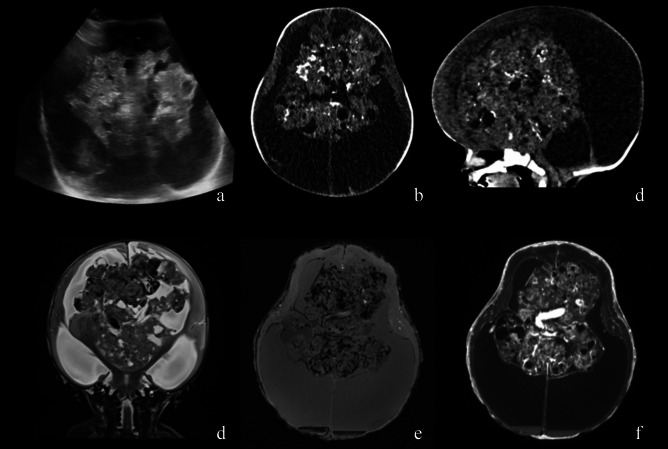
*Mature teratoma*. Neonatal US (**a**), CT (**b**, **c**) and MRI (**d**–**f**) demonstrating a heterogeneous midline intraventricular mass containing calcification, fat and solid components causing severe mass effect with hydrocephalus and diffuse brain edema. Prominent vascular structures are also evident within the lesion. Brain tissue is severely compressed and displaced at the periphery. Brain sulci and basal cisterns are partially effaced

These fusion genes lead to the transcription of chimeric TRK proteins, and it can result in the constitutive activation of the tyrosine kinase domain, leading to uncontrolled cell growth. Thus, new drugs such as NTRK inhibitors may be promising in treating these specific tumour subtypes [31, 36, 37].

A mutation that has been detected in both LGG and HGG is the BRAF mutation, and the use of BRAF inhibitors have been shown to be effective both in terms of cytoreduction and symptoms control [31, 38, 39].

Among low-grade tumours, there are subependymal giant cell astrocytomas (SEGAs), rare brain tumours that primarily affect individuals with a genetic condition known as tuberous sclerosis complex (TSC). Treatment of SEGAs involves a combination of surgical resection and targeted medical therapy with MTOR inhibitors such as everolimus that inhibits the mTOR pathway which is overactive in TSC and contributes to tumour growth [40, 41].

A case of ganglioglioma in differential diagnosis with SEGA is shown in Fig. 4.

**Fig. 3:**
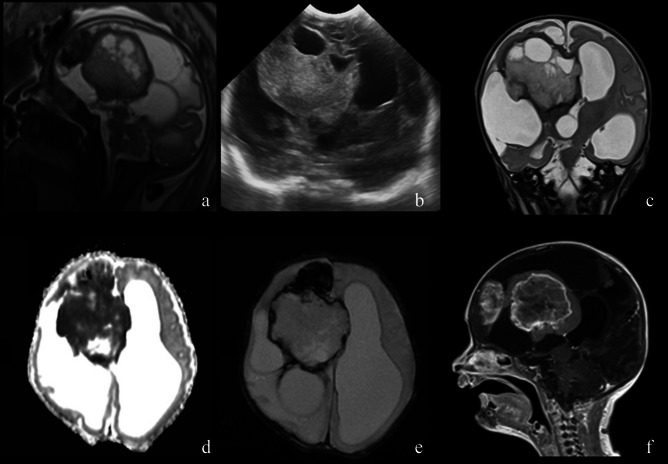
*Congenital Infant-Type Hemispheric Glioma* in a newborn. Foetal US at 35 weeks (sagittal plane, **a**), and neonatal US (coronal plane, **b**) shows a large solid-cystic and haemorrhagic mass in the right lateral ventricle. MRI images (**c**–**f**) confirm the solid-cystic hypercellular mass with blood products and heterogeneous enhancement that obliterates the right foramen of Monro causing compression and contralateral displacement of the third ventricle and severe hydrocephalus. Molecular analysis revealed NTRK1 Fusion

Choroid plexus tumours are divided into papillomas and carcinomas. In contrast to the more common papillomas that grow slowly and are generally benign, carcinomas are more aggressive and have the potential to invade nearby structures in the brain (Fig. 5) [42].

**Fig. 4:**
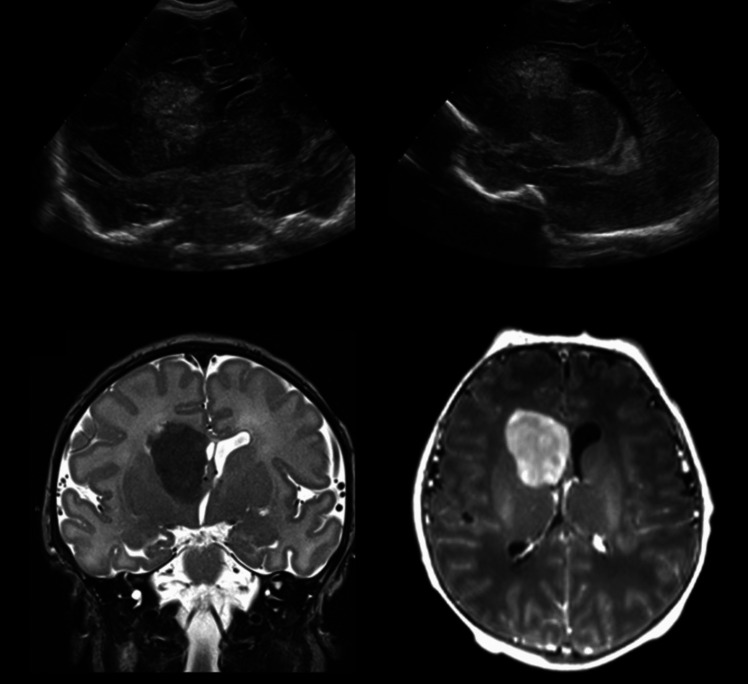
A conspicuous hyperechoic mass in close proximity to the right foramen of Monro was noted at Neonatal brain US performed soon after birth (upper row). At MRI, the mass displayed homogeneous hypointensity on T2 in stark contrast to the largely unmyelinated brain. Contrast enhancement was intense and homogeneous. Differential diagnoses at imaging included a SEGA (although no other features of TSC were noted) and a glioma. Histology revealed a *Ganglioglioma* (WHO grade 1)

When an infant is diagnosed with a choroid plexus carcinoma, especially if there is a family history of cancer, neonatologists have to consider an underlying CPS, such as Li-Fraumeni syndrome [43].

Embryonal tumours are by far the least frequent. If we consider the molecular landscape, congenital medulloblastomas are almost all SHH-activated and are often associated with Gorlin syndrome. Additionally, if ATRT is diagnosed under 12 months of age, a genetic study is required because the patient is most likely germline mutated (RTPS1 or RTPS2) [44].

Finally, less common histologies include craniopharyngiomas and ependymomas [31].

## Therapy

**Fig. 5:**
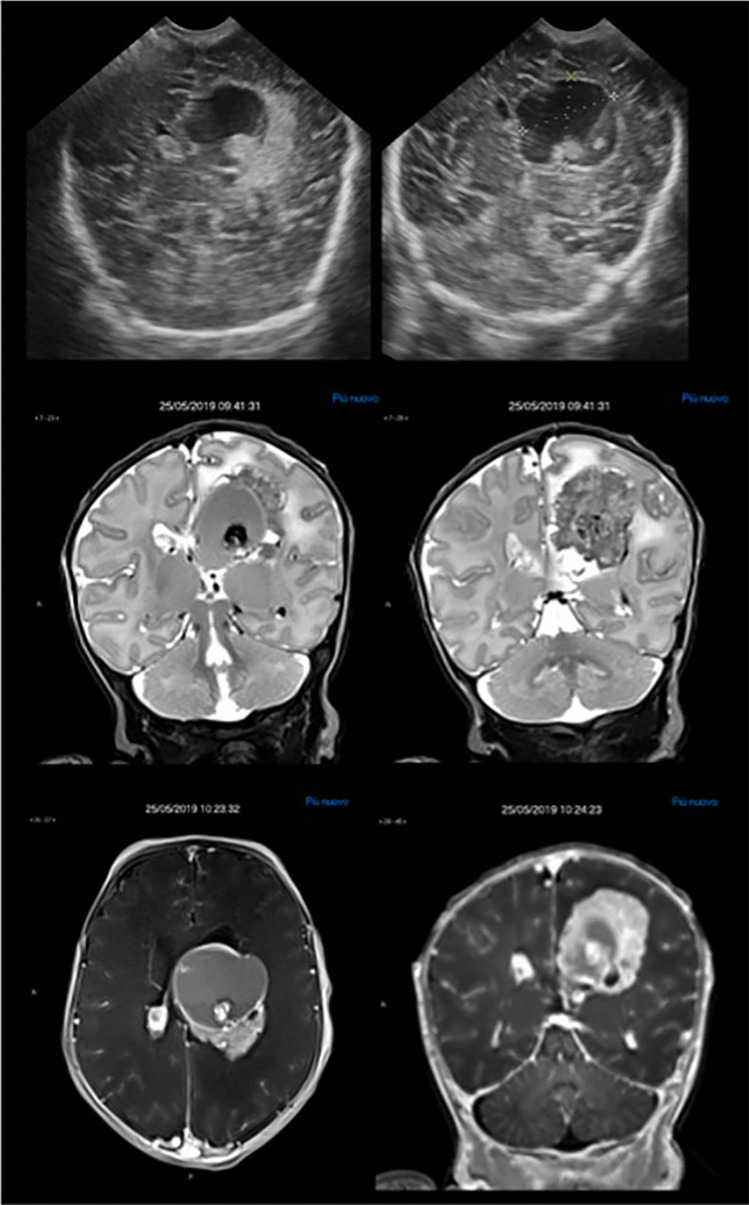
*Choroid plexus carcinoma* in a newborn. Neonatal brain US images show a hyperechoic, heterogeneous intraventricular mass with blood-fluid levels expanding the left lateral ventricle (upper row). A vascular solid-cystic mass with ill-defined margins, blood products and surrounding brain edema is confirmed at MRI (lower rows). Enlargement of the choroid plexus of the lateral ventricles due to both infiltration and congestion is also noted

Tumours of the CNS in newborns are rare but they are serious conditions that require specialised medical care. Treatment approaches for these tumours can vary depending on the type of tumour, its location, and the overall health of the neonate (Fig. 5).

### Surgery

In almost all cases, surgical resection is the primary treatment for neonatal CNS tumours, aiming for a gross total resection. Moreover, surgery also offers histological specimens allowing a more accurate diagnosis. The extent of surgical resection directly correlates with prognosis and the overall outcome.

The type and approach of neurosurgery must be discussed in a multidisciplinary team considering the anamnestic history and clinical characteristics of the infant (gestational age, birth weight, symptoms, etc.), as well as the radiological characteristics of the tumour.

Although surgery is the main prognostic element, in some cases it may not be possible to achieve a total resection. Instead, a single biopsy of the lesion may be chosen, and if necessary, a two-stage surgery may be performed.

It is important to know that many neonatal tumours present with the symptoms of hydrocephalus that often are immediately life threatening to the child rather than the tumour itself. Fortunately, there are several surgical options to deal with acute hydrocephalus such as external ventricular drain, ventriculoperitoneal shunt and third ventriculostomy [45].

### Adjuvant treatment

The two main modalities available for the postoperative treatment of CNS tumours are chemotherapy and radiotherapy. However, radiotherapy is avoided under three years of age because of the severe sequelae (long-term cognitive and development deficits, endocrine dysfunction, secondary neoplasms in the CNS) [46]. Therefore, the only adjuvant therapy is chemotherapy, which is used to treat certain types of neonatal tumours such as medulloblastomas, ATRTs, LGG, malignant gliomas and even infantile ependymoma. Due to the fragile nature of a neonate's developing brain, chemotherapy agents need to be carefully selected to minimise potential long-term neurocognitive deficits.

### Targeted therapies

Given the limited therapeutic strategies for these patients, new targeted agents that specifically target molecular or genetic abnormalities in some neonatal CNS tumours are being validated. As mentioned above, it is important to define the molecular profile of these tumours in order to identify the possibility of using target therapies.

### Supportive care

Neonates with CNS tumours may require extensive supportive care, including pain management, nutrition support, and management of side effects from treatments.

## Prognosis

Neonates with CNS tumours have a poor prognosis with overall survival of less than 30% [47]. The most common outcome is death within five years of the diagnosis [45].

As mentioned above, outcome is related to several factors such as the size and location of the tumour, directly related to the extent of surgical resection, the histologic type, the higher frequency of metastasis at the diagnosis and the limited use of adjuvant treatment to avoid severe sequelae such as secondary neoplasms in the CNS, endocrine dysfunction and potential long-term neurocognitive deficits that are reported in 40–100% of long-term survivors, probably related to vascular lesions or demyelination, which may be observed after these therapies [19]. Hence the importance of molecular characterization of these neoplasms in order to identify new therapeutic targets and thus improve the current poor prognosis of these tumours.

Because of the long-term sequelae, survivors of CNS tumours require follow-up care in terms of regular neurological and neurodevelopmental examination to identify and manage any persistent deficits, endocrinological evaluations to monitor growth, puberty, and hormonal balance, especially in tumours affecting the pituitary gland or hypothalamus, and periodic evaluations of vision and hearing to detect and manage any damage [48]. Regular screening and monitoring are also important for the early detection of potential secondary cancers, especially in the presence of a cancer predisposition syndrome.

Therefore, individualized treatment plans should be developed based on the survivor's specific medical and oncological history and health needs.

## Ethical implications

When talking about neonatal tumours, a chapter dedicated to the ethical impact of the disease cannot be excluded.

Once a neoplasm is diagnosed in the prenatal or neonatal period, a multidisciplinary interview with both expectant parents must be organised in order to provide the most complete and exhaustive counselling possible, informing them of the treatment options and prognosis, as well as the potential risks for both mother and foetus [18].

Given the good prognosis of many types of neonatal tumours, it is important for the neonatologist to rely on expert advice to strike the right balance between risks and benefits course.

## Conclusions

Neonatal CNS tumours are rare and differ in their clinical presentation, location, histological characteristics, neuroradiological features, and prognosis from tumours occurring later in childhood. Their prognosis is usually poor above all because of the limited therapeutic options. For this reason, it is important to improve the molecular characterization in order to identify new targeted agents and therefore new therapeutic strategies.

Moreover, it is crucial to emphasise that the management of neonatal CNS tumours should be individualised, taking into consideration the specific type of tumour, its location, the overall health of the neonate and the potential long-term risks and benefits of each treatment option. In this regard, it is essential to recognize these pathologies in the prenatal period in order to create a multidisciplinary team of neonatologists, obstetricians, paediatric neurosurgeons, oncologists and radiologists ready to reduce the risk of maternal and foetal complications, providing the best possible treatment while minimising the risk of long-term complications, especially considering the vulnerable nature of the neonatal brain.

## Data Availability

No datasets were generated or analysed during the current study.

## Abbreviations

ALK: Anaplastic Lymphome Kinase
APC: Adenomatous Polyposis Coli
ATM: Ataxia Telangiectasia Mutated
ATRT: Atypical Teratoid Rhabdoid Tumors
BRAF: V-raf murine sarcoma viral oncogene homolog B1
CMV: Cytomegalovirus
CNS: Central Nervous System
CPS: Cancer Predisposition Syndrome
CREBBP: CREB-binding protein
CT: Computed Tomography
DICER1: Endoribonuclease Dicer 1
DROSHA: Drosha Ribonuclease III
EP300: E1A-aasociated protein p300
FANCA: Fanconi Anemia Complementation Group A
HGG: High Grade Glioma
LGG: Low Grade Glioma
MB: Medulloblastoma
MET: Mesenchymal Epithelial Transition
MLH1: Human MutL Homolog 1
MRI: Magnetic Resonance Imaging
MSH: Human MutS Homolog
MTOR: Mammalian Target Of Rapamycin
NF: Neurofibromatosis
NTRK: Neurotrophic Tyrosine Receptor Kinase
PMS2: PMS1 Homolog 2, Mismatch Repair System Component
PTCH: Protein Patched Homolog
PTEN: Phosphatase And Tensin Homolog
RB: Retinoblastoma
RTPS: Rhabdoid Tumor Predisposition Syndrome
SEGA: SubEpendymal Giant cell Astrocytoma
SHH: Sonic Hedgehog
SMARCA4: SWI/SNF Related, Matrix Associated, Actin Dependent Regulator of Chromatin, Subfamily A, Member 4
SMARCB1: SWI/SNF Related, Matrix Associated, Actin Dependent Regulator of Chromatin, Subfamily B, Member 1
TOR: Target Of Rapamycin
TP53: Tumor Protein p53
TRK: Tropomyosin Receptor Kinase
TSC: Tuberous Sclerosis Complex
TSE: Turbo Spin Echo
US: Ultrasound
VHL: Von Hippel Lindau
WHO: World Health Organization
